# P-1480. *Staphylococcus aureus* Susceptibilities Among Patients Who Use Illicit Fentanyl in Philadelphia, Pennsylvania

**DOI:** 10.1093/ofid/ofae631.1650

**Published:** 2025-01-29

**Authors:** Drew Dickinson, Steve Saw, Lauren Dutcher, Christina Maguire, Adrienne Terico, Margaret Lowenstein, Sonal Patel

**Affiliations:** Hospital of the University of Pennsylvania, Philadelphia, Pennsylvania; Hospital of the University of Pennsylvania, Philadelphia, Pennsylvania; University of Pennsylvania Perelman School of Medicine, Philadelphia, Pennsylvania; Penn Presbyterian Medical Center, Philadelphia, PA; Pennsylvania Hospital, Philadelphia, Pennsylvania; University of Pennsylvania, Philadelphia, Pennsylvania; Hospital of the University of Pennsylvania, Philadelphia, Pennsylvania

## Abstract

**Background:**

Patients who use drugs are at high risk for *Staphylococcus aureus* infection. Within the Northeastern United States, fentanyl has become the prominent substance in the illicit opioid supply. We developed a *S. aureus* antibiogram among patients who use fentanyl (PWUF) presenting with acute *S. aureus* skin or skin structure infections (SSSI) in Philadelphia, Pennsylvania.

Table 1

**Figure:**
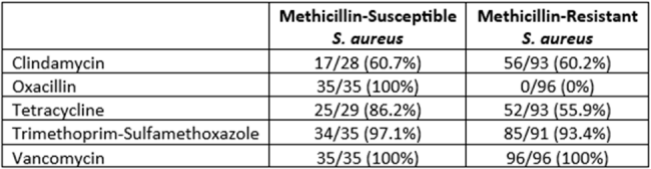

In vitro susceptibility of S. aureus clinical isolates from the wounds of people who use fentanyl. Susceptibilities presented as a proportion of total isolates tested for each antibiotic.

**Methods:**

In this retrospective, multi-center study, patients presenting to one of four Penn Medicine hospitals with an acute *S. aureus* SSSI and illicit fentanyl use within the previous year were included. Susceptibilities of *S. aureus* isolated from skin and skin structure cultures were described among the PWUF cohort and compared to the health system’s outpatient wound antibiogram. Opioid withdrawal parameters were compared between patients who left the hospital prior to and after the availability of *S. aureus* susceptibilities.

Table 2

**Figure:**
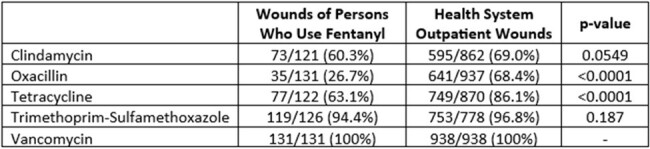

In vitro susceptibility of S. aureus clinical isolates from the wounds of people who use fentanyl compared to S. aureus isolated from wounds of all outpatients in the health system. Susceptibilities presented as a proportion of total isolates tested for each antibiotic.

**Results:**

Among 131 *S. aureus* isolates from 131 PWUF presenting with an acute SSSI, 35/131 (26.7%) were susceptible to oxacillin, 73/121 (60.3%) were susceptible to clindamycin, 77/122 (63.1%) were susceptible to tetracycline, and 119/126 (94.4%) were susceptible to trimethoprim-sulfamethoxazole (Table 1). PWUF displayed significantly reduced susceptibility to oxacillin and tetracycline compared to the health system’s outpatient wound *S. aureus* antibiogram (Table 2). Seventy (53.4%) patients were discharged prior to the availability of *S. aureus* susceptibilities, 46 (65.7%) of which were patient-directed discharges. Compared to patients discharged prior to the availability of susceptibilities, a greater proportion of patients discharged after the reporting of susceptibilities were administered buprenorphine or methadone in the hospital (82.0% vs 51.4%, p< 0.001).

**Conclusion:**

High rates of non-susceptibility to clindamycin and tetracycline suggest these agents should not be prescribed as empiric therapy for acute *S. aureus* SSSI in PWUF. PWUF would benefit from joint management by infectious diseases and addiction medicine experts to prevent premature discharge and ensure prescription of active therapy.

**Disclosures:**

**Christina Maguire, PharmD**, Viiv: Advisor/Consultant

